# Fatal Aortoesophageal Fistula after Esophagectomy for Esophageal Cancer: A Single-Center Retrospective Analysis

**DOI:** 10.5761/atcs.oa.26-00037

**Published:** 2026-04-23

**Authors:** Lianzheng Zhao, Zhen Zhang, Huijiang Gao, Guodong Shi, Jiangshan Ai, Yucheng Wei

**Affiliations:** 1 Department of Thoracic Surgery, The Affiliated Hospital of Qingdao University, Qingdao, China; 2 Department of Thoracic Surgery, Shanghai General Hospital, Shanghai Jiao Tong University School of Medicine, Shanghai, China

**Keywords:** esophageal cancer, esophagectomy, aortoesophageal fistula, anastomotic leak, massive hemorrhage

## Abstract

**Purpose:**

Aortoesophageal fistula (AEF) after esophagectomy is a rare but catastrophic complication, often occurring after anastomotic leakage and mediastinal contamination. Evidence regarding fatal trajectories and rescue failure points remains limited.

**Methods:**

We performed a retrospective, single-center study among esophageal cancer patients who developed AEF after esophagectomy between 2013 and 2024. Cases were identified from institutional databases and mortality records, followed by manual screening. Clinical course, antecedent complications, diagnostic workup, rescue interventions, and causes of death were summarized descriptively.

**Results:**

Among 5543 esophagectomies, 17 patients (0.31%) developed AEF: 16 fatal (94.1%) and 1 survivor (5.9%). All were male, median age 63 years. Anastomotic leak occurred in 16 patients (94.1%), all with mediastinal infection. Sentinel bleeding preceded hemorrhage in 14 patients (82.4%); the median interval to fatal hemorrhage was 1 day.

**Conclusions:**

Post-esophagectomy AEF is highly lethal. Sentinel bleeding represents a critical intervention window. Prompt recognition and multidisciplinary escalation may enable survival even in high-risk patients.

## Introduction

Esophageal cancer is the ninth most common malignant tumor worldwide. In China, it ranks seventh, resulting in nearly 200000 deaths each year.^1)^ Esophagectomy remains the most definitive curative approach for esophageal cancer, including upfront resection for early-stage patients and resection after neoadjuvant therapy for locally advanced patients. Esophagectomy is a challenging and highly invasive surgery that makes postoperative recovery difficult for patients and often leads to a series of complications.^2)^ Among these, there is a rare but fatal complication known as aortoesophageal fistula (AEF).^3)^

AEF is a rare but almost always fatal aorto-digestive tract communication disease, which is caused by the formation of an abnormal channel between the thoracic aorta and the esophagus, leading to sudden upper gastrointestinal hemorrhage and often accompanied by mediastinal contamination and severe infection.^4,5)^ AEF can be classified into primary and secondary types: the former is mostly associated with thoracic aortic aneurysms/dissections, penetrating foreign bodies, malignant tumors, radiation injury, or infectious lesions; the latter often occurs after thoracic aortic surgery or endovascular treatment (such as thoracic endovascular aortic repair [TEVAR]), with mechanisms including long-term mechanical erosion by artificial materials/stents, local necrosis due to endoleaks, graft infection, and ischemia of surrounding tissues.^6)^ The classic clinical presentation is the “Chiari triad”: chest pain or dysphagia−small amount of bleeding as a precursor—fatal massive hemorrhage after a brief interval.^7)^ Although enhanced computed tomography angiography (CTA) and endoscopy are of great value in locating the bleeding and suggesting the fistula, the examination window is extremely limited in cases of continuous bleeding or hemodynamic instability.

Especially worth emphasizing is that AEF following radical esophagectomy for esophageal cancer is one of the rarest but most catastrophic secondary types of AEF.^5)^ Although its incidence is extremely low, the survival rate is very low once it occurs. Its mechanism of occurrence is usually due to the continuous local suppuration and tissue necrosis after anastomotic fistula, gradually eroding the thoracic aorta and its branches, and eventually breaking into the esophagus or gastric tube.^8)^ Clinically, it often presents as sudden hematemesis in postoperative patients, or sudden massive bleeding through the nasogastric tube or mouth, and rapidly leads to death. Due to the rarity of this complication, most existing studies are case reports or small sample reviews, suggesting the need for a systematic summary of related cases and the proposal of more operational diagnosis and treatment procedures.^9)^ Therefore, this study retrospectively reviewed the course and data of patients with this complication after esophageal cancer surgery in a single center in China.

## Materials and Methods

We conducted a retrospective, single-center observational study of patients who developed AEF after esophagectomy for esophageal cancer between January 2013 and December 2024. This study was approved by the institutional review board of the Affiliated Hospital of Qingdao University on June 24, 2025 (Approval No. QYFY WZLL 30266). The requirement for informed consent was waived due to the retrospective nature of the study, consistent with the ethical standards of our institution.

To restrict the study population, we utilized the Yidu Cloud platform (Yidu Cloud Technology Ltd., Beijing, China), a big-data intelligence platform that integrates electronic medical records data from our centers. The system possesses the ability to retrieve and screen medical records. The Yidu Cloud platform was used to search for patients who were diagnosed with esophageal cancer in the hospital. Subsequently, the cases were manually screened. The following inclusion and exclusion criteria were used to select the study population. The inclusion criteria were as follows: (1) Patients diagnosed with esophageal cancer and who underwent esophagectomy. (2) Patients who developed clinically significant postoperative bleeding potentially attributable to AEF. The exclusion criteria were as follows: (1) Patients with other serious diseases or complications, where the death case discussion could not clearly determine the cause of death. (2) Patients with severely incomplete clinical data, making it impossible to determine the treatment process.

Collected variables included patient demographics, tumor characteristics, treatment details (neoadjuvant therapy, surgical approach, anastomotic location), postoperative course (anastomotic leak, mediastinal infection [mediastinitis defined as the presence of purulent drainage from mediastinal drains, positive microbial cultures from mediastinal specimens, or radiographic evidence of mediastinal fluid collections or abscess formation on CT in the setting of clinical signs of infection {fever, leukocytosis, elevated inflammatory markers}], re-interventions [defined as any surgical, endoscopic, or radiologic procedure performed to address postoperative complications after the index esophagectomy, including drainage procedures, leak repairs, stent placements, or vascular interventions]), and specifics of the AEF event (timing of sentinel and massive hemorrhage, diagnostic modalities, resuscitation, and hemostatic interventions).

Analyses were descriptive. Continuous variables were summarized as median (interquartile range) or mean (standard deviation), and categorical variables as n (%). Time intervals were summarized with medians and ranges. Analyses were performed using SPSS 25.0 (IBM, Armonk, NY, USA).

## Results

During the study period, 5543 esophagectomies were performed. Seventeen patients (0.31%) developed clinically confirmed AEF: 16 fatal cases (94.1%) and 1 survivor (5.9%). All patients were male, with a median age of 63 years (range: 42–74). Tumors were most frequently located in the middle and middle-lower thoracic esophagus. All patients underwent esophagectomy, with intrathoracic anastomosis in 15 (88.2%) and cervical anastomosis in 2 (11.8%) cases. Postoperative adjuvant therapy was administered to 7 patients (41.2%). Baseline characteristics are summarized in **Table 1**, with comparative data between fatal cases and the survivor.

**Table 1:** Baseline characteristics and surgical details of patients with aortoesophageal fistula (n = 17)

Characteristic	All AEF patients (n = 17)	Fatal cases (n = 16)	Survivor (n = 1)
Sex, male, n (%)	17 (100%)	16 (100%)	1
Age (years), median (range)	63 (42–74)	64 (42–74)	54
Tumor location, n (%)			
Cervical	1 (5.9%)	1 (6.3%)	0
Upper thoracic	0 (0%)	0 (0%)	0
Middle thoracic	6 (35.3%)	6 (37.5%)	0
Middle-lower thoracic	5 (29.4%)	5 (31.3%)	0
Lower thoracic	4 (23.5%)	4 (25.0%)	1
Cardia	1 (5.9%)		
Anastomotic location, n (%)			
Intrathoracic (supra-/infra-aortic)	15 (88.2%)	14 (87.5%)	1
Cervical	2 (11.8%)	2 (12.5%)	0
Adjuvant therapy, n (%)	7 (41.2%)	7 (43.8%)	0
Postoperative anastomotic leak, n (%)	16 (94.1%)	15 (93.8%)	1
Postoperative mediastinal infection, n (%)	17 (100%)	16 (100%)	1

AEF, aortoesophageal fistula

All patients developed major complications prior to AEF. An anastomotic leak was confirmed in 16 patients (94.1%), with a median time to leak of 24 days postoperatively (range: 1–9125 days). For the survivor, an anastomotic leak was inferred from endoscopic findings of diffuse bleeding at the anastomosis on postoperative day 4. All 17 patients developed subsequent mediastinal infection.

Sentinel bleeding (defined as a minor, self-limited episode of hematemesis, hemoptysis, or bloody chest tube output) occurred in 14 patients (82.4%). The median time from sentinel bleed to catastrophic hemorrhage was 1 day (range: 0–60 days). Catastrophic hemorrhage presented as massive hematemesis or sudden hemodynamic collapse. Diagnostic evaluation was severely limited by the emergent nature of the condition; only 5 patients (29.4%) underwent CTA during the event, which was suggestive of the diagnosis in 2 cases.

Management universally involved aggressive resuscitation, including massive transfusion. Targeted hemostatic interventions were attempted in 12 patients (70.6%): endoscopic therapy (n = 5), TEVAR (n = 4), open surgical repair (n = 2), and transarterial embolization (n = 1). Among fatal cases, 2 had prolonged intervals from sentinel bleed to death after surgical repair or embolization, but all ultimately succumbed. The clinical course after the onset of massive hemorrhage was precipitous. The median time from the diagnosis of AEF (based on the catastrophic bleed) to death was 1 day (range: 0–125 days) for fatal cases; the survivor was discharged alive. Key timelines and interventions are detailed in **Table 2**.

**Table 2:** Key timelines and interventions in the clinical trajectory of aortoesophageal fistula

Item	All AEF patients (n = 17)	Fatal cases (n = 16)	Survivor (n = 1)
Time from surgery to anastomotic leak (days), median (range)	24 (1–9125)	24 (1–9125)	4
Sentinel bleeding documented, n (%)	14 (82.4%)	13 (81.3%)	Yes
Interval from sentinel bleed to massive hemorrhage (days), median (range)	1 (0–60)	1 (0–60)	<1 day (h)
CTA performed during/pre-hemorrhage, n (%)	5 (29.4%)	5 (31.3%)	No
Targeted hemostatic intervention attempted, n (%)	12 (70.6)	11 (68.8%)	Yes
Endoscopic hemostasis	5	4	1
TEVAR	4	4	0
Open surgical repair	2	2	0
Transarterial embolization	1	1	0
Time from AEF diagnosis (massive bleed) to death (days), median (range)	1 (0–125)	1 (0–125)	0

CTA, computed tomography angiography; TEVAR, thoracic endovascular aortic repair; AEF, aortoesophageal fistula

## Discussion

In this series of 17 post-esophagectomy AEF patients, the lethal trajectory followed a consistent catastrophic pathway: anastomotic leak, mediastinal infection, aortic erosion, sentinel bleeding, and rapid progression to exsanguination.^4)^ However, with 16 fatalities (94.1%) and only 1 survivor (5.9%), our findings underscore the extreme lethality of this complication while demonstrating that survival is possible in rare instances with prompt recognition and aggressive intervention.

Sentinel bleeding is defined as a minor, self-limited episode of hematemesis, hemoptysis, or bloody chest tube output. The high incidence of sentinel bleeding (82.4%) aligns with other reports on AEF, underscoring its role as a critical clinical sign.^10)^ The median interval of only 1 day from sentinel bleed to fatal hemorrhage in deceased patients highlights the narrow therapeutic window. Given the 94.1% mortality, one might reasonably question whether sentinel bleeding should be regarded primarily as a sign of death rather than an actionable warning signal. In the vast majority of cases, sentinel bleeding heralds an inexorable progression to fatal hemorrhage, and the window for effective intervention is exceedingly brief. This interpretation is clinically important: it sets appropriate expectations for patients and families while reinforcing the urgency of response. The single survivor in our series provides a critical counterpoint, demonstrating that while sentinel bleeding is overwhelmingly associated with mortality, it is not uniformly fatal. In a minority of cases, the window for intervention, though narrow, can be successfully exploited. Therefore, we propose that sentinel bleeding in post-esophagectomy patients with mediastinal infection should be regarded as a sign of impending death unless promptly and aggressively addressed.

The survivor’s course illuminates predisposing clinical conditions. This 54-year-old patient with hypertension and uremia on hemodialysis experienced sentinel bleeding on postoperative day 4 and survived precisely because this warning sign triggered immediate escalation: bedside endoscopy, intensive care unit transfer, mechanical ventilation, and continuous renal replacement therapy. His comorbidities—long-standing hypertension (potentially contributing to aortic wall vulnerability) and uremia requiring hemodialysis (associated with platelet dysfunction and impaired healing)—likely increased susceptibility to vascular erosion. The rapid rise in inflammatory markers (procalcitonin; C-reactive protein) suggests an infectious trigger contributing to anastomotic breakdown. The low rate of pre-hemorrhage CTA (29.4%) in our series underscores potential systems delays. For any post-esophagectomy patient with mediastinal infection, even trivial bleeding must be considered a vascular emergency warranting urgent aortic imaging and multidisciplinary response.^11)^ Most available evidence for post-esophagectomy AEF consists of case reports and small series. Our study complements existing literature by characterizing a consistent fatal phenotype centered on anastomotic leak–mediastinitis, while also demonstrating through a survivor and existing successful cases^6,11,12)^ that timely, coordinated intervention can alter outcomes. Although staged approaches combining endovascular hemostasis and subsequent definitive source control are used, fatal outcomes remain common—especially when diagnosis is delayed, hemorrhagic shock is advanced, or mediastinal sepsis is uncontrolled.

The data presented here carry a more important clinical implication: once sentinel bleeding has occurred, outcomes are rarely salvageable. Therefore, the focus of clinical efforts should shift to earlier phases of the disease process—specifically, the prevention of progression from anastomotic leak to aortic erosion. In our study, all patients who developed AEF had antecedent anastomotic leak with subsequent mediastinal infection. This observation aligns with existing literature demonstrating that persistent, uncontrolled mediastinal sepsis is the essential substrate for aortic wall erosion.^13,14)^ In clinical practice, early identification and aggressive management of anastomotic leak and mediastinal infection are therefore critical to prevent catastrophic progression. Patients with suspected leaks should undergo prompt CT evaluation to assess the extent of mediastinal contamination and guide drainage strategies. Early, aggressive drainage—whether percutaneous, endoscopic, or surgical—may reduce the infectious burden and potentially interrupt the cascade leading to aortic erosion. This preventive perspective is supported by studies showing that timely source control in mediastinal infections is associated with improved outcomes.^12)^

**Fig. 1:**
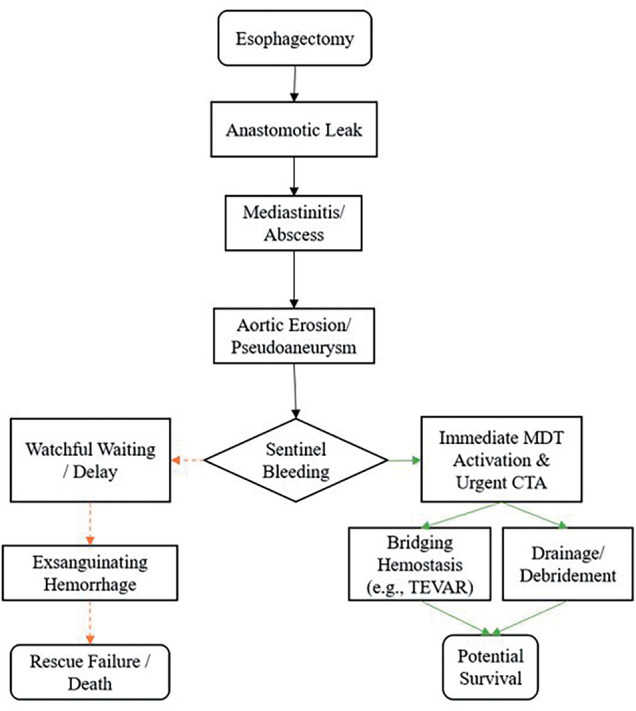
Progression and clinical management of fatal aortoesophageal fistula. CTA, computed tomography angiography; TEVAR, thoracic endovascular aortic repair; MDT, multi disciplinary team

Based on these considerations, we propose a 2-stage clinical approach. The first stage focuses on early prevention: for patients with confirmed anastomotic leak and mediastinal infection, early CT evaluation should be performed to assess the anatomic relationship between the infectious focus and the aorta. Aggressive drainage of mediastinal collections, broad-spectrum antibiotics targeting enteric organisms, and optimization of nutritional and metabolic status are essential to prevent tissue necrosis and aortic erosion. Patients with persistent infection despite conservative measures may warrant earlier surgical or endoscopic re-intervention. The second stage addresses emergency response: if sentinel bleeding occurs despite optimal preventive measures, an immediate protocol-driven response is required. As shown in **Fig. 1**, this involves activating a multidisciplinary team (thoracic surgery, vascular surgery, interventional radiology, critical care), proceeding to urgent thoracic CTA if hemodynamically feasible, and based on imaging, jointly deciding on an intervention plan. If a pseudoaneurysm or definite fistula is identified, emergency TEVAR combined with planned mediastinal exploration and debridement should be considered. If hemorrhage is already uncontrollable, the risks and potential benefits of a heroic thoracotomy must be weighed.^15)^

This study has several limitations. Its retrospective, single-center design introduces potential selection and information biases. Some AEF cases with mild presentations may have been missed. Diagnosis in the survivor, while strongly supported by clinical context and endoscopic findings, was not confirmed by CTA or autopsy. The single survivor limits generalizability of comparative analyses.

## Conclusions

Post-esophagectomy AEF is highly lethal, with a clinical trajectory consistently following anastomotic leak, mediastinal infection, and aortic erosion. Sentinel bleeding portends imminent death. However, one survivor demonstrates that prompt recognition and multidisciplinary escalation can alter this outcome. Sentinel bleeding in high-risk patients should therefore be regarded as a sign of impending death unless urgently addressed, warranting immediate CTA and intervention without delay.

## Declarations

### Availability of data and materials

All data generated or analyzed during this study are included in this published article.
